# Efficacy and Safety of Adherence to dl-3-n-Butylphthalide Treatment in Patients With Non-disabling Minor Stroke and TIA—Analysis From a Nationwide, Multicenter Registry

**DOI:** 10.3389/fneur.2021.720664

**Published:** 2021-09-22

**Authors:** Zefeng Tan, Yin Zhao, Wanyong Yang, Shenwen He, Yan Ding, Anding Xu

**Affiliations:** ^1^Department of Neurology, the First Affiliated Hospital, Jinan University, Guangzhou, China; ^2^Department of Neurology, Shun De Hospital of Jinan University, Guangzhou, China

**Keywords:** minor stroke, dl-3-n-butylphthalide, TIA, efficacy, modified rankin scale, non-disabling

## Abstract

**Background:** Dl-3-n-Butylphthalide (NBP) has the potential to improve clinical outcomes in acute ischemic stroke patients by improving collateral circulation. We aimed to evaluate the efficacy and safety of NBP in patients with non-disabling minor ischemic stroke and transient ischemic attack (TIA).

**Methods:** The BRIDGE (the observation study on clinical effectiveness of NBP on patients with non-disabling ischemic cerebrovascular disease) is a prospective registry to monitor the efficacy and safety of NBP therapy in acute non-disabling ischemic stroke or high-risk TIA. Non-disabling minor ischemic stroke patients within 48 h were enrolled across 51 stroke centers in China. We divided patients into NBP compliance or non-compliance groups according to their adherence to NBP. The primary outcome was the favorable functional outcome at 90 days, defined as a modified Rankin scale (mRS) <2.

**Results:** Between 10th October 2016 and 25th June 2019, 3,118 patients were included in this analysis. In multivariable analysis, after adjusting for common risk factors and demographic factors, NBP-compliance group has a higher proportion of favorable functional outcome (92.1 vs. 87.4%, adjusted odds ratio 2.00, 95% confidence interval, 1.50–2.65), and a higher stroke recurrence rate (2.40 vs. 0.31%, adjusted odds ratio 8.86, 95% confidence interval, 3.37–23.30) than the NBP-non-compliance group. There was no significant difference in death and intracranial hemorrhage rate between the two groups. In subgroup analysis, patients with National Institutes of Health Stroke Scale (NIHSS) scores from 3 to 5 who complied to NBP therapy had a higher rate of favorable functional outcomes than the NBP-non-compliance group. [88.82 vs. 76.21%, adjusted odds ratio 2.52 (1.81–3.50), adjusted interaction *P* = 0.00].

**Conclusion:** In non-disabling minor ischemic stroke or TIA patients, compliance with NBP therapy led to better 90-day functional outcomes despite a higher risk of recurrence, and this effect seems to be stronger in patients with NIHSS scores of 3–5. Further large randomized, double-blind controlled studies to analyse the association between NBP and functional outcome is warranted in the coming future.

## Introduction

Studies have reported that minor stroke and high-risk TIA (ABCD2 score ≥ 4) may have a high risk of early stroke recurrence and a more unsatisfactory clinical outcome than expected (1–4). Although treatment of minor stroke and high-risk TIA has improved, the risk of stroke recurrence within 90 days of stroke onset has been reported to be as high as 10–20% (1–4). Randomized, double-blind controlled studies based on Chinese populations have reported a 90-day fatal or disability incidence of 6.03% and a 90-day stroke recurrence rate of 9.6% in patients with minor stroke [National Institutes of Health Stroke Scale (NIHSS), 0–3] or high-risk TIA (5).

Evaluating collateral circulation at an early stage will facilitate the selection of intravenous thrombolysis and endovascular therapy (6–8). More importantly, a robust collateral flow will compensate for blood supply in the ischemic area, reduce core infarct volume and revive the penumbra zone.

Dl-3-n-butylphthalide (NBP) is a synthetic compound based on l-3-n-butylphthalide extracted from the seeds of Apium graveolens Linn. Synthesized NBP has been approved to treat ischemic stroke patients with medium and small infarct foci in China (9). NBP improved collateral circulation by restoring the diameter of meningeal micro-arteries in the ischemic area and promoting blood vessel formation in mice (10, 11). Results of several multicenter randomized, double-blind, placebo-controlled trials evaluating oral NBP in patients with acute ischemic stroke showed significant improvements in neurological deficits and activity of daily living scales in the NBP -treated group compared with the placebo-controlled group, with a good safety profile (9, 12), and accepted by the Chinese guidelines for acute ischemic stroke (13). However, real-world, large-scale clinical studies of NBP therapy in patients with non-disabling minor stroke and high-risk TIA are still lacking.

This study aimed to analyze the efficacy and safety of NBP in patients with Non-disabling minor ischemic stroke and high-risk TIA.

## Methods

### The BRIDGE Registry

The BRIDGE (the observation study on clinical effectiveness of NBP on patients with non-disabling ischemic cerebrovascular disease) is a prospective registry that was initiated in 2018 to monitor efficacy and safety of NBP therapy in acute non-disabling ischemic stroke (NIHSS ≤ 5 on admission) or high-risk TIA (ABCD2 score ≥ 4 on admission). For the BRIDGE registry, specific information on timing and dosing of NBP, antiplatelet and statin therapy, and other stroke risk factors was added to the standard questionnaire. Data presented were obtained by standardized questionnaires in the 51 participating hospitals, medical history (prior coronary interventions, congestive heart failure, diabetes mellitus, renal insufficiency), biochemical test results, stroke subtype, and brain imaging. The clinical research forms available for the study were centralized at NCRC-ND (The China National Clinical Research Center for Neurological Diseases) and recorded into the Electronic Data Capture System [EDC 1.0, Jianhe Jiuzhou (Beijing) Technology Co.]. All data were analyzed centrally at NCRC-ND, China. Local monitoring and audits of the data have been performed in 10% of randomly selected patients. The ethics committee has approved the registry study. The local ethics committee has approved the registry of each center. Trial registration: http://www.chictr.org.cn/ Identifier: ChiCTR-OPC-16008069.

### Patient Selection

All patients with non-disabling ischemic stroke (NIHSS ≤ 5 on admission) or high-risk TIA (ABCD2 score ≥ 4 on admission) within 48 h of onset and treated with NBP were included on an intention-to-treat basis. Exclusion criteria include: (1) intracranial hemorrhage or vascular malformation, tumor, abscess, or other common non-ischemic brain diseases (e.g., multiple sclerosis); (2) iatrogenic stroke; (3) patients receiving early recanalization therapy (intravenous thrombolysis or endovascular thrombectomy); (4) pre-morbid mRS score >2; and (5) severe multi-organ failure; (6) patients with severe mental disorders and dementia; (7) patients who are pregnant or nursing or planning to become pregnant. All patients underwent cranial MRI during hospitalization to determine cerebral infarction diagnosis and exclude mimic stroke.

### Procedure and NBP Medication Compliance

**Figure 1:**
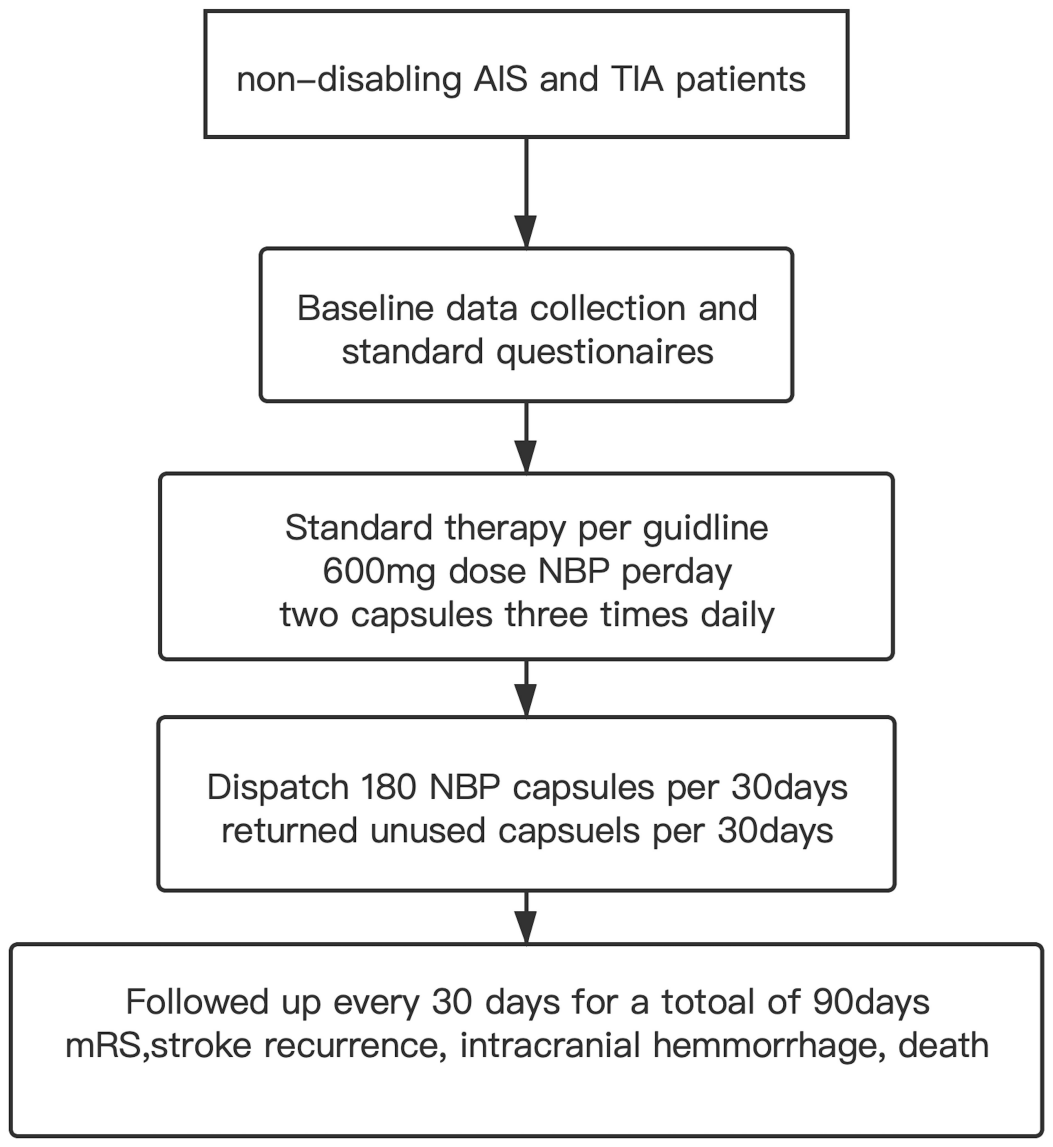
Flow progress diagram.

The 600 mg dose of NBP was taken as two capsules three times daily, each containing 100 mg of NBP. We invited all patients to take part in the study. Physicians contacted patients to obtain informed consent. All patients who agreed to participate in the study were instructed to take standard dose of NBP for 90 days and asked to complete a questionnaire at baseline; they were followed up every 30 for 90 days (Figure 1: flow progress diagram). For each month follow-up period, the patient received a pack of 180 capsules, and they were instructed to return the unused capsules at each visit. Nurses interviewed the patients. Patients were asked to record the duration of any lapses, the altered dosages that may have been taken, and the frequency of omitting any single dose. The answers were recorded in a completed CRF form for double-checking of the unreturned pills by follow-up personnels. Compliance with medication was calculated as the number of nonreturned capsules divided by six times the number of days in the follow-up period. The daily capsule count was expressed as a percentage of the scheduled dose (14). NBP medicine compliance was defined as taking 80% or more of the prescribed NBP doses (15). We monitored the patient's adverse reactions in accordance with the usual follow-up requirements, and the patient's liver and kidney function monitoring is carried out during baseline and routine follow-up.

### Sample Size

Assuming the proportion of good functional outcome (mRS0–1) in NBP-compliance group is 85%, and in NBP-non compliance group is 80%, power of 0.90, and a level of 0.05, we required 1,209 participants per group. To account for possible dropouts of 10%, we increased the sample to 2,688.

### Statistical Methods

All statistical analyses were performed using SAS version 9.4, and continuous variables are expressed as mean and standard deviation or median and quartiles.

We present data as absolute numbers or percentages. The Pearson–Fisher χ^2^ test compared the frequencies of categorical variables in the two treatment groups. The Mann–Whitney–Wilcoxon test compared continuous variables.

We compared the proportion of stroke recurrence at 90 days between the two groups using Fisher's exact probability test or χ^2^ test. We calculated HRs and 95% confidence intervals using single-factor and multifactor Cox regression.

We compared the proportion of favorable outcomes (mRS ≤ 1) between the two groups using single-factor and multifactor regression analysis.

We used single and multivariable Cox regression to compare the proportion of death and intracranial hemorrhage between the two groups at 90 days and calculate HR and 95% confidence intervals. A propensity score for NBP-compliance treatment was created to adjust for baseline differences between NBP-compliance and NBP-non-compliance subjects. A logistic regression model with compliance -NBP treatment as the outcome was used to generate propensity scores for all subjects. Variables that are potential confound compliance for NBP were included in three models; model 1 (age and sex); model 2 (age, sex, history of hypertension, body mass index, history of diabetes, history of dyslipidemia, smoking, alcohol consumption, admission NIHSS score, and TOAST types); modle 3 (propensity score). All hypotheses were tested using a two-sided test with α = 0.05. *P* ≤ 0.05 was considered statistically significant.

## Results

Between 10th October 2016 and 25th June 2019, 4,592 consecutive patients with minor acute ischemic stroke or high-risk TIA within 48 h of onset were included in the BRIDGE registry. A total of 152 patients were excluded because of incomplete information, of which 12 were missing important baseline information (age, sex, height, and weight), and 140 were missing important follow-up information (mRS scores at discharge and day 90). A total of 2,966 cases were entered into the analytical dataset based on the definition of the analytical dataset (Supplementary Figure 1), of which there were 1,042 cases in the NBP-compliance group and 1,924 cases in the NBP-non-compliance group. Table 1 shows the baseline characteristics in the two groups.

**Table 1:** Baseline characteristics between NBP compliance and NBP-non-compliance group.

**Variate**	**ALL**	**NBP-non-compliance group (*n* = 1,924)**	**NBP compliance group (*n* = 1,042)**	***P***
Age, years		65.00 (56.00–73.00)	64.00 (55.00–72.00)	0.0779
Male	1,935 (65.23%)	1,234 (64.14)	701 (67.27)	0.0868
Onset to admission time (h)		10.12 (3.00–24.00)	10.01 (2.52–24.00)	0.0283
Diabetes, *N* (%)	763 (25.72)	486 (25.26)	277 (26.58)	0.4311
Hypertension, *N* (%)	1,954 (65.88)	1,270 (66.01)	684 (65.64)	0.8412
Dyslipidemia, *N* (%)	2,707 (91.27)	1,762 (91.58)	945 (90.69)	0.4129
Stroke history, *N* (%)	110 (3.7)	70 (3.64)	40 (3.84)	0.7827
Smoke, *N* (%)	735 (24.78)	469 (24.38)	266 (25.53)	0.0175
Atrial fibrillation, *N* (%)	80 (2.70)	66 (3.43)	20 (1.92)	0.0192
Coronary heart disease, *N* (%)	374 (12.61)	229 (11.90)	145 (13.92)	0.1148
Previous anti-hypertension, *N* (%)	1,415 (47.71)	941 (48.91)	474 (45.49)	0.0751
Previous antiplatelet, *N* (%)	593 (20.00)	371 (19.28)	222 (21.31)	0.1886
NIHSS, median (IQR)	2.00 (1.00–4.00)	2.00 (1.00–3.00)	2.00 (1.00–4.00)	0.0003
**Current medication therapy**				
Antiplatelet, *N* (%)	2,751 (92.75)	714 (92.13)	2,037 (92.97)	0.4371
Anticoagulant, *N* (%)	240 (8.10)	61 (7.87)	179 (8.17)	0.7932
Stain therapy, *N* (%)	2,447 (82.50)	625 (80.65)	1,822 (83.16)	0.1135
Anti-hypertension, *N* (%)	1,297 (43.73)	359 (46.32)	938 (42.81)	0.0903

*Values expressed as no./total no. (%) unless otherwise indicated. mRS, modified rankin scale; NIHSS, National Institutes of Health Stroke Scale*.

Of the 2,966 patients included in this study, 2,751 (92.7%) were treated with antiplatelet therapy, of which 1,229 (43.80%) were treated with aspirin only, 389 (13.11%) with clopidogrel only, 1,233 (41.57%) with dual antiplatelet, and 215 (7.25%) were not treated with any antiplatelet agents.

The mean age of the NBP-non-compliance and the NBP compliance group was 65.00 (56.00–73.00) and 64.00 (55.00–72.00) years. The median onset time from onset to hospital admission was 10.12 (3.00–24.00) h and 10.01 (2.52–24.00) h for the NBP-non-compliance group and the NBP compliance group, respectively. The median NIHSS score at admission was 2 for each of the two groups. The remaining baseline characteristics are detailed in Table 1.

### Primary Outcomes

**Figure 2:**
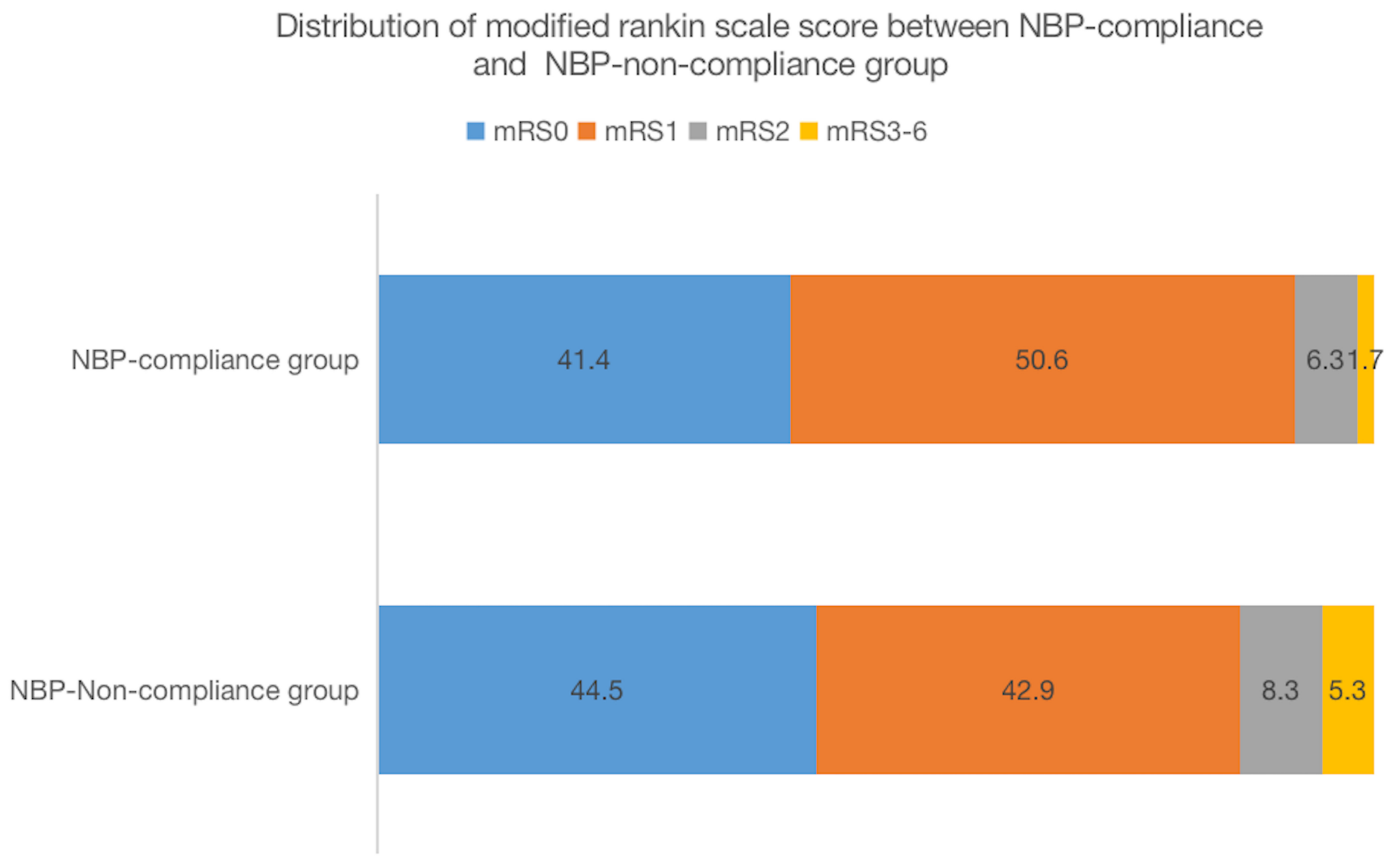
Distribution of modified rankin scale score between NBP-compliance and NBP-non-compliance group.

The proportion of patients with a favorable 90-day functional outcome (mRS 0–1) was higher in the NBP-compliance group (92.05%, 949 cases) than in the NBP-non-compliance group (87.42%, 1,626 cases) (OR = 1.67, 95% CI, 1.28–2.17, and *p* = 0.0002). In logistic regression model 1, after adjusting for age and sex, there was a significant difference in the proportion of favorable functional outcomes between the two groups (OR = 1.63, 95% CI, 1.25–2.13, and *p* = 0.0003). In the logistic regression model 2, after adjusting for age, sex, history of hypertension, body mass index, diabetes, dyslipidemia, smoking, alcohol consumption, TOAST types, and admission NIHSS score, the proportion of favorable functional outcomes remained significantly different between the two groups (OR = 2.00, 95% CI, 1.50–2.65, and *p* < 0.0001; Table 2). After adjusting for propensity score in model 3, we found the similar results between the two groups [OR = 1.97 (1.5–2.59), *p* < 0.0001]. The distribution of the modified Rankin scale scores between NBP-compliance and NBP-non-compliance group was shown in Figure 2.

**Table 2:** Outcome in the bridge trial according to compliance of nbp treatment.

	**NBP-non -compliance group**	**NBP compliance group**	**No adjusted**	***P***	**Model 1**	***P***	**Model 2**	***P***
	***n* (%)**	***n* (%)**	**HR/OR (95% CI)**		**HR/OR (95% CI)**		**HR/OR (95% CI)**	
mRS (0–1) 90-day	1,626 (87.42)	949 (92.05)	1.67 (1.28–2.17)	0.0002	1.63 (1.25–2.13)	0.0003	1.86 (1.41–2.45)	<0.0001
Stroke recurrence	6 (0.31)	25 (2.40)	7.78 (3.19–18.95)	<0.0001	7.65 (3.14–18.66)	<0.0001	7.85 (3.22–19.18)	<0.0001
Intracranial hemmorrhage	0 (0.00)	3 (0.29)	–	–	–	–	–	–
Death	4 (0.21)	0 (0.00)	–	–	–	–	–	–

*Model 1: Adjusted for age and sex; Model 2: Adjusted for age, sex, history of hypertension, body mass index, past history: history of diabetes, history of dyslipidemia, smoking, alcohol consumption, and admission NIHSS score*.

### Key Secondary and Other Efficacy Outcomes

The recurrence rate of ischemic stroke within 90 days was significantly higher in the NBP-compliance group than the NBP-non-compliance group (2.4 vs. 0.31%, OR = 7.78, 95% CI, 3.19–18.95, and *P* < 0.0001; Table 2). The incidence of death within 3 months was 0 cases (0.00%) in the NBP-compliance group and 4 cases (0.21%) in the NBP-non-compliance group (Table 2). Among all TIA patients, only one patient had stroke recurrence, and this patient was in the NBP-compliance group (*p* = 0.30).

### Bleeding Events

The primary safety endpoint major bleeding occurred rarely and was not different between the groups (0.29% NBP-compliance group and 0.0% NBP-non-compliance group).

### Subgroup Analysis

The high proportion of 90-day favorable functional outcomes in the NBP-compliance group was consistent across the major subgroups, with interaction *p* > 0.05, except that the 90-day favorable functional outcome was better in the NIHSS (3–5) subgroup (88.82%, 453 cases) than in the NIHSS (0–2) subgroup (95.20%, 496 cases) (OR = 2.52, 95% CI, 1.81–3.50, and interaction *p* = 0.00), after adjustment for age, sex, history of hypertension, body mass index, diabetes, dyslipidemia, smoking, alcohol consumption, and admission NIHSS score (Table 3).

**Table 3:** Subgroup analysis on favorable functional outcome at 90 days (mRS 0–1).

	**NBP-non-compliance group, *n* (%)**	**NBP compliance group, *n* (%)**	^*^Adjusted OR (95% CI)	***P* for interaction**
**Sex**				
Male	680 (88.43)	418 (92.68)	2.06 (1.32–3.19)	0.4671
Female	372 (86.11)	183 (90.59)	1.59 (0.89–2.84)	
**Age**				
<60	343 (90.03)	215 (95.98)	3.01 (1.36–6.64)	0.1580
≥60	709 (86.46)	386 (89.98)	1.63 (1.10–2.42)	
**BMI**				
<25 kg/m^2^	1,052 (87.59)	601 (92.04)	1.90 (1.34–2.70)	0.8304
≥25 kg/m^2^	574 (87.10)	348 (92.06)	1.79 (1.13–2.84)	
**Hypertension**				
No	384 (87.67)	221 (94.04)	2.36 (1.25–4.47)	0.4183
Yes	668 (87.55)	380 (90.91)	1.75 (1.15–2.67)	
**Diabetes**				
No	818 (88.82)	449 (92.77)	1.80 (1.18–2.72)	0.8211
Yes	234 (83.57)	152 (89.94)	2.21 (1.14–4.31)	
**Dyslipidemia**				
No	69 (82.14)	52 (94.55)	3.92 (0.97–15.80)	0.2053
Yes	983 (88.00)	549 (91.81)	1.77 (1.23–2.54)	
**Smoking**				
Yes	799 (87.23)	445 (90.63)	1.60 (1.09–2.34)	0.0586
No	253 (88.77)	156 (96.30)	3.96 (1.55–10.09)	
**Alcohol consumption**				
No	807 (87.24)	438 (90.50)	1.61 (1.10–2.36)	0.0590
Yes	245 (88.77)	163 (96.45)	4.26 (1.67–10.85)	
**Admission NIHSS score**				
0	296 (97.69)	115 (96.64)	0.53 (0.15–1.92)	0.1151
1–5	1,330 (85.42)	834 (91.45)	1.82 (1.38–2.39)	
**Admission NIHSS**				
0–1	621 (96.43)	259 (95.57)	0.79 (0.38–1.61)	0.0185
2–5	1,005 (82.65)	690 (90.79)	2.04 (1.52–2.73)	
**Admission NIHSS**				
0–2	1,043 (95.25)	496 (95.20)	0.94 (0.58–1.55)	0.0016
3–5	582 (76.21)	453 (88.82)	2.52 (1.81–3.50)	

* *Adjusted for age, sex, body mass index, past history: history of hypertension, history of diabetes, history of dyslipidemia, smoking, alcohol consumption, and admission NIHSS score*.

## Discussion

The principal result of this study is that compliance to NBP-treatment increased 90 days favorable functional outcome rate by 4.63% compared with the NBP-non-compliance patients (OR = 1.86, 95% CI, 1.41–2.45) in non-disabling ischemic stroke or TIA patients, suggesting that the regular use of NBP in patients with minor stroke and high-risk TIA may improve functional outcomes and reduce disability. A *post-hoc* subgroup analysis raises the hypothesis that patients with NIHSS scores 3–5 may achieve a higher proportion of favorable functional outcomes at 90 days among patients with NIHSS scores 0–5. This result suggests that NBP treatment may reduce the rate of disability in patients with potentially disabling minor strokes.

### NBP Compliance and Functional Outcome

Our study suggested a recent substantial-high proportion of favorable functional outcomes (86.8%, 2,577/2,966) in patients with non-disabling minor ischemic stroke or TIA. However, cohort studies suggested about 30% of patients with minor stroke had a functional disability at 90 days after stroke despite nonsevere deficits at presentation (16, 17). The most likely reason for the discrepancy between previous cohort studies and the BRIDGE trial is the different inclusion criteria. Patients requiring intravenous thrombolysis and emergency revascularization were excluded from the present study; thus, many patients with potentially disabling stroke and large vessel stenosis or occlusion were excluded. Data from the United States showed that more than one-half of acute ischemic stroke hospitalizations had minor deficits, accounting for 4 of every 10 intravenous thrombolysis therapy (IVT) and 1 of every 10 mechanical thrombectomy treatments (18). A nationwide study in the United States, the excellent functional outcome only occurred in 48.2% of all minor strokes who underwent IVT or endovascular therapy (EVT) (18). The exclusion of EVT and IVT minor ischemic stroke or TIA patients from the present study might contribute to the substantial-high proportion of favorable functional outcomes. Only non-disabling minor stroke patients, rather than all minor stroke patients, were enrolled in this study was another contributor to the substantial-high favorable functional outcome rate. Our study suggested treatment with NBP, in addition to other standard treatments, may result in an additional good functional outcome in non-disabling ischemic stroke patients with no indication for EVT or IVT. This finding may be relevant to the pharmacological mechanism of NBP.

### Mechanism of NBP in Stroke

NBP is a synthetic compound that has been approved for the treatment of ischemic stroke in China. The underlying mechanisms of the NBP-treatment efficacy have been reported in multiple studies. Animal experiments have shown that NBP can significantly improve the collateral circulation: rapidly opening the secondary collateral circulation, restoring the diameter of meningeal micro-arteries in the ischemic area and increasing blood flow velocity (19); establishing the tertiary collateral circulation, promoting the expression of VEGF and promoting vascular neovascularization (20). Recent animal studies have shown that daily intranasal NBP treatment can stimulate neurogenesis and angiogenesis in mice with ischemic strokes (21). NBP might address different pathophysiological functions, including anti-oxidation, anti-inflammatory, anti-apoptosis, antithrombotic, and mitochondrial protection for acute ischemic stroke treatment (22).

Furthermore, another randomized clinical trial included 170 patients and found that NBP significantly increased circulating levels of endothelial progenitor cells in acute ischemic stroke patients and improved clinical outcomes (mRS at 90 days) (12).

### NBP Compliance and Stroke Recurrence

Data from the CNSR study (the China National Stroke Registry) in China (from 2007 to 2008) showed that the 3-month recurrence rate of cerebrovascular disease (included ischemic stroke, intracranial hemorrhage, and subarachnoid hemorrhage) among patients with minor stroke and TIA was 9.8% (23). However, the proportion of patients with minor stroke or TIA who had a 90-day recurrence was only 1.04% (31/2,966) in this study cohort, lower than in previous studies (23). This discrepancy could be attributed to several possible reasons. First of all, previous reports of stroke risk after TIA is highest in the first 7 days and in patients without immediate treatment (11.0%; 95% CI, 8.6–13.5%) (24). Over the past two decades, new treatment strategies and early management of TIA and minor strokes have significantly reduced stroke recurrence rates (25). The lowest risks were seen in studies of emergency treatment in specialist stroke services [0.9% (95% CI, 0.0–1.9)] (26–29) and the highest risks in population-based studies without urgent treatment [11.0% (8.6–13.5)] (30–32). There was substantial heterogeneity between studies. Furthermore, in a most recent systematic review and meta-analysis of 206,455 patients in 68 unique studies during 5 decades, the subsequent risk of ischemic stroke was 4.7% within 90 days and the incidence was gradually decreased by the time of trials, among the study population recruited before 1999 was significantly higher (25). Second of all, the definition of stroke recurrence varies between studies, resulting in different incidences of observed stroke recurrence. One study showed a 90-day recurrence rate of 20/180 for patients with minor stroke and TIA, but a real new stroke (infarction at a new site) of only 2/180 was found at 30-day follow-up using MRI (33). Thirdly, the dual antiplatelet therapy in the present study (41.57%) and the combined treatment with NBP may also have contributed to the lower stroke recurrence rate. The present study found that the 90 days ischemic stroke recurrence rate was significantly higher in the NBP-compliance group than in the NBP-non-compliance group. However, due to the low overall event rate and the short follow-up period, we cannot rule out the possibility of an incidental finding. A large-scale study with a more extended follow-up period is needed to explore this finding.

### Medication Adherence

In the present study, 80% was used as the cutoff value of NBP drug compliance. Previous observational studies that evaluated the relationship between medication compliance and outcome used 80–100% as an indicator of high medication compliance (15), and found that it was associated with better blood pressure control (34) and blood sugar control (35). Furthermore, pharmacy supplement data and patient self-reports are the most commonly used methods of compliance assessment which is similar to the present study (15).

Despite a higher rate of recurrences, patients in the compliance group had a greater proportion of functional outcomes than patients in the non-compliance group. This discrepancy is due to the fact that patients in the compliance group with recurrent strokes still have a good functional outcome after 90 days. In this study, both the NBP-compliance and non-compliance groups had recurrent stroke cases of 25 and 6, respectively. However, the proportion of 90-day good functional outcomes in the NBP-compliance group was 64.00% (16/25), compared with 33.33% (2/6) in the NBP-non-compliance group (Supplementary Table 1). We also noticed that in the NBP-compliance group, 36.00% (9/25) of stroke recurrence occurred in patients with baseline NIHSS0–1 and TIA in the NBP-non-compliance group, and 50.00% (3/6) in NBP compliance group. The interaction between baseline NIHSS score and 90-day functional outcomes might be related to the relatively high proportion of good functional outcomes in the NBP compliance group.

The strengths of this study are mainly that, (1) the data collected prospectively in 51 cities in China represent the Chinese population with non-disabling minor stroke and TIA. (2) This study was the largest data analysis based on the efficacy analysis of treatment with NBP in the Chinese non-disabling minor stroke and TIA population. (3) In patients who are not suitable for intravenous thrombolysis or acute endovascular thrombectomy therapy, NBP treatment might improve functional outcomes at 90 days, providing a potential alternative treatment for non-disabling minor stroke and TIA patients.

The limitations of this study are, (1) that the patients enrolled were not classified for stroke etiology, such as the proportion of patients with symptomatic large vessel occlusion, and minor ischemic strokes with large vessel occlusions may be associated with higher stroke recurrence and progression. (2) The present study used 90-day mRS as the primary outcome, and there is a possibility that a longer follow-up period may have identified more stroke recurrence. (3) The treatment strategy for both groups were determined by the physician based on the patient's condition. Potentially disabling patients or patients with large vessel occlusion have been excluded due to administration of thrombolytic therapy or endovascular thrombectomy, so the present study did not reflect the characteristics of the overall minor stroke and TIA patients cohort. Another shortcoming of this study is that patients who were not compliant with NBP may also be non-compliant with other medications. Adherence to antithrombotic, lipid-lowering, and antihypertensive drugs was ensured during follow-up in our study, but adherence to glucose-lowering drugs was not followed up in this study.

## Conclusion

In this real-world experience, patients who complied with NBP therapy gained better short-term functional outcomes than those who did not, and the efficacy might even be more in those patients with NIHSS score from 3 to 5. Further large scale randomized controlled trial to explore the association between NBP therapy and non-disabling minor acute ischemic stroke or TIA outcomes is warranted in the coming future.

## Data Availability Statement

The original contributions presented in the study are included in the article/Supplementary Material, further inquiries can be directed to the corresponding author/s.

## Ethics Statement

The studies involving human participants were reviewed and approved by the Medical Ethics Council of the First Affiliated Hospital of Jinan University. The patients/participants provided their written informed consent to participate in this study.

## Author Contributions

ZT and AX conceived and designed the study. YZ, SH, and YD performed the study. YZ and ZT analyzed the data. ZT wrote the paper. AX gave suggestions how to design the study, edit the results, and write the manuscript. All authors contributed to the article and approved the submitted version.

## Conflict of Interest

The authors declare that the research was conducted in the absence of any commercial or financial relationships that could be construed as a potential conflict of interest.

## Publisher's Note

All claims expressed in this article are solely those of the authors and do not necessarily represent those of their affiliated organizations, or those of the publisher, the editors and the reviewers. Any product that may be evaluated in this article, or claim that may be made by its manufacturer, is not guaranteed or endorsed by the publisher.

## Supplementary Material

Supplementary Figure 1 Trial flow diagram.

Supplementary Table 1 Proportion of 90 day good functional outcomes in patients with stroke recurrence.
