# Corrigendum: Efficacy and Safety of Adherence to dl-3-n-Butylphthalide Treatment in Patients With Non-disabling Minor Stroke and TIA—Analysis From a Nationwide, Multicenter Registry

**DOI:** 10.3389/fneur.2021.800494

**Published:** 2021-11-23

**Authors:** Zefeng Tan, Yin Zhao, Wanyong Yang, Shenwen He, Yan Ding, Anding Xu

**Affiliations:** ^1^Department of Neurology, the First Affiliated Hospital, Jinan University, Guangzhou, China; ^2^Department of Neurology, Shun De Hospital of Jinan University, Guangzhou, China

**Keywords:** minor stroke, dl-3-n-butylphthalide, TIA, efficacy, modified rankin scale, non-disabling

In the original article, there was an error. The Results section contained an error regarding the number of subjects in the compliance and non-compliance NBP groups. The number of non-compliance patients was mistakenly listed as the number of compliance patients in the results section. Also, the number of patients in the non-compliance group was taken as the number of patients in the compliance group.

A correction has been made to Results, paragraph 1, lines 8 to 12, page 3.

A total of 2,966 cases were entered into the analytical dataset based on the definition of the analytical dataset (**Supplementary Figure 1**), of which there were 1,042 cases in the NBP-compliance group and 1,924 cases in the NBP-non-compliance group.

In the original article, there was an error. The second error is related to subgroup analysis. There was a discrepancy between the subgroup analyses described in this article and those shown in **Table 3** of NIHSS0-2 and NIHSS3-5. Our initial statistical analysis section contained an error related to our subgroup analysis, which we corrected, and then updated our table, but we did not update the Abstract and Subgroup Analysis sections.

A correction has been made to the Abstract, Results, lines 10 to 11.

…[88.82 vs. 76.21%, adjusted odds ratio 2.52 (1.81–3.50), adjusted interaction *P* = 0.00].

A correction has been made to Subgroup Analysis, lines 5 to 7, page 4.

…subgroup (88.82%, 453 cases) than in the NIHSS (0–2) subgroup (95.20%, 496 cases) (OR = 2.52, 95% CI, 1.81–3.50, and interaction *p* = 0.00).

The authors apologize for this error and state that this does not change the scientific conclusions of the article in any way. The original article has been updated.

## Publisher's Note

All claims expressed in this article are solely those of the authors and do not necessarily represent those of their affiliated organizations, or those of the publisher, the editors and the reviewers. Any product that may be evaluated in this article, or claim that may be made by its manufacturer, is not guaranteed or endorsed by the publisher.

