# Supplementary figures and images for: Efficacy and Safety of Adherence to dl-3-n-Butylphthalide Treatment in Patients With Non-disabling Minor Stroke and TIA—Analysis From a Nationwide, Multicenter Registry
Source: Front Neurol. 2021 Sep 22;12:720664. doi: 10.3389/fneur.2021.720664 (PMC8492907; doi:10.3389/fneur.2021.720664)

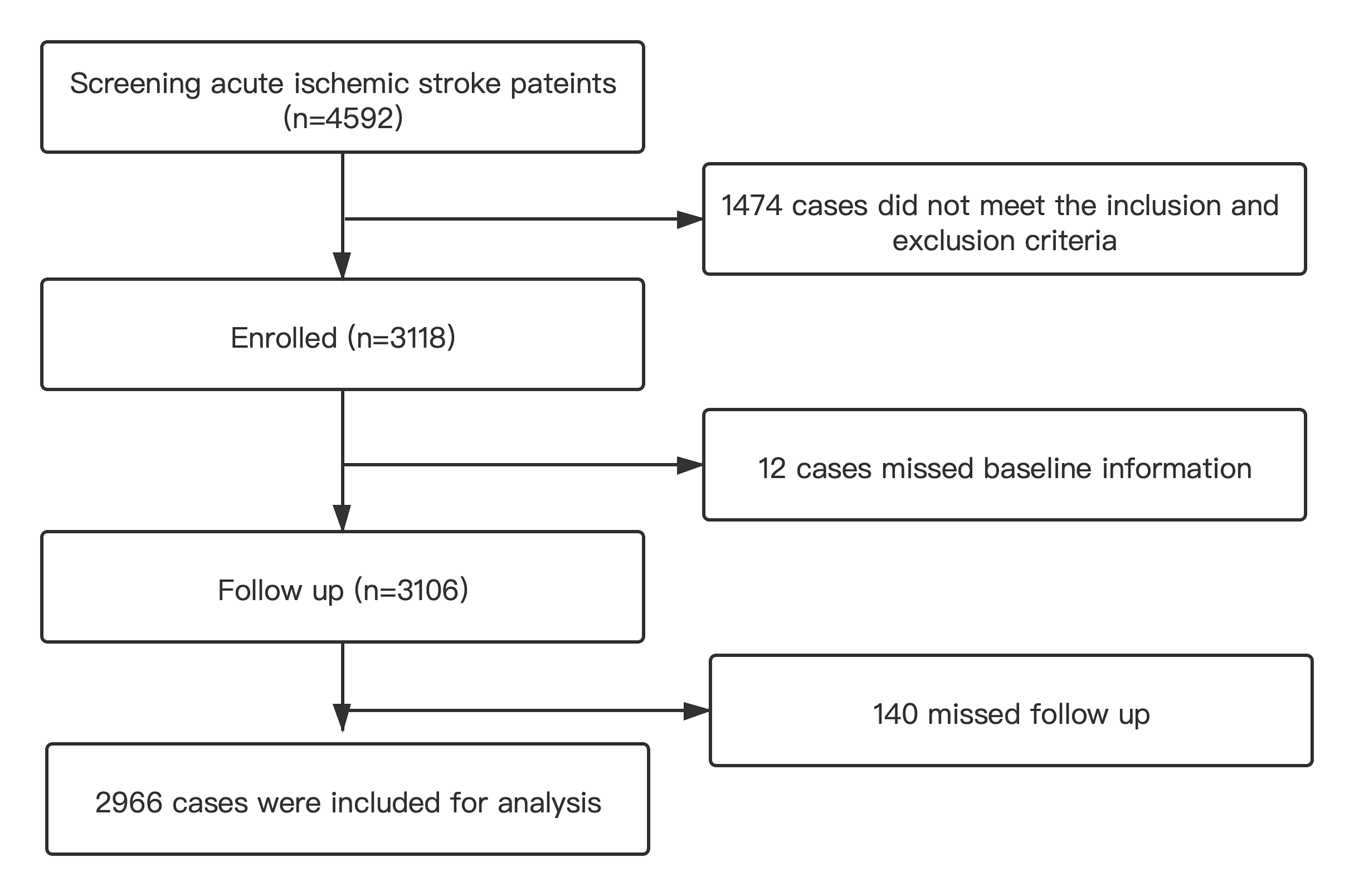

Supplement: Supplementary Figure 1 — Trial flow diagram. [file Image_1.JPEG]
