# Supplementary material for: Efficacy and Safety of Adherence to dl-3-n-Butylphthalide Treatment in Patients With Non-disabling Minor Stroke and TIA—Analysis From a Nationwide, Multicenter Registry
Source: Front Neurol. 2021 Sep 22;12:720664. doi: 10.3389/fneur.2021.720664 (PMC8492907; doi:10.3389/fneur.2021.720664)
Supplement: Supplementary Table 1 — Proportion of 90 day good functional outcomes in patients with stroke recurrence. [file Table_1.DOCX]

Supplement file I Proportion of 90 day good functional outcomes in patients with stroke recurrence

|  | NBP Non-compliance group | NBP Compliance group |
| --- | --- | --- |
| mRS (0-1) 90-day | 2 (33.33) | 16 (64.00) |
| mRS >1 90-day | 4 (66.67) | 9 (36.00) |
